# The Appearance of 4-Hydroxy-2-Nonenal (HNE) in Squamous Cell Carcinoma of the Oropharynx

**DOI:** 10.3390/molecules25040868

**Published:** 2020-02-16

**Authors:** Antonia Jakovčević, Kamelija Žarković, Danica Jakovčević, Zoran Rakušić, Drago Prgomet, Georg Waeg, Suzana Borović Šunjić, Neven Žarković

**Affiliations:** 1 Clinical Hospital Centre Zagreb, Clinical Department of Pathology and Cytology, School of Medicine, University of Zagreb, Kispaticeva 12, 10000 Zagreb, Croatia; kamelijazarkovic@gmail.com; 2 Department of Pathology, Clinical Hospital “Sv. Duh”, Ul. Sveti Duh 64, 10000 Zagreb, Croatia; danica.jakovcevic@gmail.com; 3 Department of Oncology, University Hospital Centre Zagreb, Kispaticeva 12, 10000 Zagreb, Croatia; zrakusic@kbc-zagreb.hr; 4 Clinic for Ear, Nose and Throat Diseases and Head and Neck Surgeries, University Hospital Center Zagreb, Kispaticeva 12, 10000 Zagreb, Croatia; dprgomet2@gmail.com; 5 Institute of Molecular Biosciences, Karl Franzens University, Humboldtstrasse 50, A-8010 Graz, Austria; georg.waeg@uni-graz.at; 6 Rudjer Boskovic Institute, Laboratory for Oxidative Stress, Bijenička cesta 54, 10000 Zagreb, Croatia; borovic@irb.hr (S.B.Š.); zarkovic@irb.hr (N.Ž.)

**Keywords:** 4-Hydroxy-2-nonenal (HNE), immunohistochemistry, lipid peroxidation, oxidative stress, protein modifications, monoclonal antibodies, squamous cell carcinoma, oropharynx

## Abstract

Tumor growth is associated with oxidative stress, which causes lipid peroxidation. The most intensively studied product of lipid peroxidation is 4-hydroxy-2-nonenal (HNE), which is considered as a “second messenger of free radicals” that binds to proteins and acts as a growth-regulating signaling factor. The incidence of squamous cell carcinoma of the oropharynx is associated with smoking, alcohol and infection of human papilloma virus (HPV), with increasing incidence world-wide. The aim of this retrospective study involving 102 patients was to determine the immunohistochemical appearance of HNE-protein adducts as a potential biomarker of lipid peroxidation in squamous cell carcinoma of the oropharynx. The HNE-protein adducts were detected in almost all tumor samples and in the surrounding non-tumorous tissue, while we found that HNE is differentially distributed in squamous cell carcinomas in dependence of clinical stage and histological grading of these tumors. Namely, the level of HNE-immunopositivity was increased in comparison to the normal oropharyngeal epithelium in well- and in moderately-differentiated squamous cell carcinoma, while it was decreasing in poorly differentiated carcinomas and in advanced stages of cancer. However, more malignant and advanced cancer was associated with the increase of HNE in surrounding, normal tissue. This study confirmed the onset of lipid peroxidation, generating HNE-protein adducts that can be used as a valuable bioactive marker of carcinogenesis in squamous cell carcinoma of the oropharynx, as well as indicating involvement of HNE in pathophysiological changes of the non-malignant tissue in the vicinity of cancer.

## 1. Introduction

**Figure 1:**
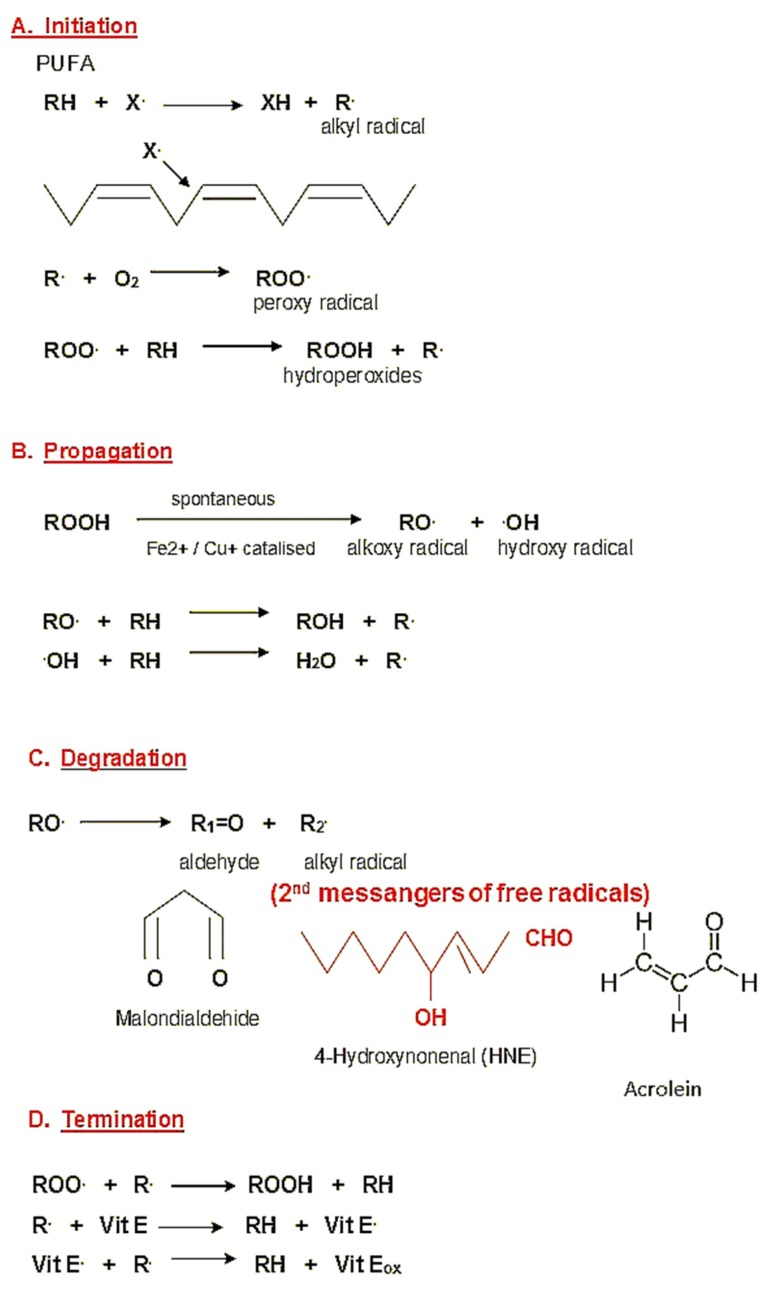
Generation of HNE as a product of lipid peroxidation. (**A**) Due to the attack of oxygen free radical X. (mostly hydroxyl radical) on the omega-6-poylunsaturade acids (PUFA), the chain-reaction of lipid peroxidation is initiated. (**B**) Lipid hydroperoxides can be spontaneously or under the influence of transition metals further metabolized generating radicals themselves. (**C**) The end products of oxidative degradation of PUFA are reactive aldehydes, among which HNE exhibits the most prominent biochemical and biomedical effects, acting as a second messenger of free radicals. (**D**) In the case of the presence of lipid-soluble antioxidants, the initial stage of lipid peroxidation can be terminated.

Oxidative stress (OS) is the process of excessive production of reactive oxygen species (ROS), notably of free radicals, playing a major role in the etiology of major human diseases, including cancer. It occurs when the anti-oxidative capacities of cells and tissues are in disbalance with ROS, which causes oxidative damage of cells. In equilibrium, free radicals are neutralized and removed by enzymatic and nonenzymatic antioxidants [1]. Excessive production of ROS induces lipid peroxidation (LPO), especially of omega-6-polyunsaturated acids, which evolves as the autocatalytic process generates highly reactive aldehydes, notably 4-hydroxy-2-nonenal (HNE), malondialdehyde (MDA) and acrolein (Figure 1) [2,3]. The most intensively studied product of lipid peroxidation is HNE, which is considered to be “the second messenger of free radicals” that affects pathophysiology of cells in various ways. High concentrations of HNE are cytotoxic and mutagenic, while in physiological processes, present at low levels, HNE acts as a signaling molecule involved in growth regulation, interacting with cytokines and regulating the expression of cellular (proto)oncogenes [2,4,5]. The cytotoxic effects of reactive aldehydes resemble the toxicity of ROS, but because of their higher chemical stability, these aldehydes can spread from the site of origin and react with major biomolecules, among which proteins appear to be the major target, even at the distant site. Because of high reactivity with biomolecules and its multiple biological effects, HNE is a popular LPO product in the field of molecular oncology.

Carcinogenesis is a multistep process, and its etiology is still not completely understood. During the past decade, permanent oxidative damage caused by overproduction of ROS and consequent biomolecular damage has been proposed to play important roles in both initiation and in promotion of cancer development [6,7]. Considering the complexity of mechanisms of carcinogenesis and of oxidative stress, cancer may be considered both as a cause and as a consequence of oxidative stress. Malignant alteration is a long-term process from normal to neoplastic cell, during which many intracellular molecular mechanisms occur, changing the structure and function of specific tumor suppressor genes or proto-oncogenes, which are also affected by HNE [8].

Oropharyngeal carcinoma refers to cancer of the tonsil, base and posterior one-third of the tongue, soft palate, and posterior and lateral pharyngeal walls. Squamous cell carcinoma (SCC) comprises over 95% of oropharyngeal cancers [9]. As its incidence is increasing, squamous oropharyngeal cancer makes 10% of all head and neck cancers or more than 52,000 new cases annually worldwide [10]. Although oropharyngeal cancer is more common among elderly people, in recent years, patients with oropharyngeal cancer have been diagnosed younger and more commonly female as human papilloma virus (HPV) infection has emerged as an important factor of its etiology [11]. Genetic predisposition also plays an important role in oropharyngeal carcinogenesis [12], while people who both drink and smoke heavily have 30 times more risk for developing oropharyngeal cancer. According to the recent World Health Organization (WHO) classification, oropharyngeal cancers can be divided into two types, HPV-positive, which are related to human papilloma virus infection, and HPV-negative cancers, which are usually linked to alcohol or tobacco use [13]. Hence, a genetic predisposition may occur as a consequence of increased mutagen sensitivity, or an inability to metabolize carcinogens or pro-carcinogens, or to repair the DNA damage [14].

Squamous cell cancer arises from a multilayered squamous epithelium or from a cylindrical epithelium after squamous cell metaplasia. Invasive growth is manifested by invasion of the basal membrane and infiltration of underlying tissue, blood vessels and nerves [15]. According to the World Health Organization (WHO), on the basis of the amount of keratin formation and the morphological appearance of the cell, squamous cell carcinoma is divided into three grades: Grade I —well differentiated, Grade II—moderately well differentiated, and Grade III—poorly differentiated epithelial neoplasm. Surface epithelium close to invasive cancer often shows premalignant changes likely to be caused by the same carcinogens [16]. The majority of tumors of the oropharynx reveal themselves most often in the late stages of the disease with metastases in the regional lymph nodes of the neck.

Patients who do not have affected lymph nodes at the time of diagnosis have an excellent prognosis while patients with affected lymph nodes have 50% less chance of 5-year survival regardless of the treatment applied [17]. Progress in understanding the genetic and molecular basis of cancer has led to the research and discovery of specific changes in oropharyngeal squamous cell carcinoma that help prevention, timely diagnosis and adequate treatment [13,18].

Complex roles of LPO in human carcinogenesis are still not sufficiently understood. Namely, LPO is generally considered to be an undesirable process of oxidative stress, which results not only in destruction of the lipids attacked by ROS, but also in generation of their cytotoxic and carcinogenic end-products, especially in the case of the LPO of omega-6-polyunsaturated fatty acids (PUFAs). Thus, recent immunohistochemistry studies have implied that LPO products like HNE and acrolein can serve even as prognostic factors [19,20]. This might be due to the concentration-dependent cytotoxic and growth regulating effects of HNE on one side and on the other on its involvement in inflammation in the tumor itself and in the surrounding non-tumor tissue [21]. The occurrence of HNE as the final product of lipid peroxidation in tumors has been well described especially for glial tumors [22]. Immunohistochemical positivity of HNE was shown to be present in astrocytic, oligodendroglial and ependymal tumors, while its expression was gradually increasing in tumor cells with higher grade and neovascularization, which can be explained by the high concentration of PUFAs, the major source of HNE in blood vessels and in cellular lipid biomembranes. In malignant variants of glial tumors, the HNE immunopositivity was moderate to strong and was diffusely distributed in tumor tissue. The HNE was expressed in endothelium and vascular walls of almost all tumor vessels and it was increased with the grade of malignancy. These findings suggest that LPO is a common process in all glial tumors generating that an increase of HNE may be associated with malignancy and neovascularization [23]. Similarly, a retrospective study of patients with breast cancer has shown the expression of HNE as the strongest in invasive breast carcinomas, while 8-OHdG expression diminished in invasive breast carcinomas compared to non-invasive carcinomas [24]. In kidney cancer, the immunopositivity of HNE was observed in cytoplasm of tumor cells but without correlation with the clinical stage of the disease, while in healthy non-malignant kidney cells, HNE-protein adducts were not found [25].

In the studies on involvement of ROS and HNE in carcinogenesis of colorectal cancer, there are contradictory findings. Kondo et al. found a positive correlation between HNE expression and disease progression [26], while Biasi and her group found significantly reduced HNE expression in colon cancer tissue compared with non-malignant colon tissue, but not in the advanced stages of the disease [27]. Since non-malignant colon tissue used by Biasi et al. was obtained from patients with Crohn’s disease, immunohistochemical findings of human colon carcinoma require better understanding of the pathophysiology of HNE in cancer and its association with chronic inflammation.

The aim of this retrospective study was to analyze the appearance of HNE-protein adducts in oropharyngeal squamous cell carcinoma and to confirm the assumed onset of oxidative stress and lipid peroxidation in its carcinogenesis. To date, no research has been published on possible involvement of HNE in cancer development in this field, hence, the results take another step forward in understanding this particular malignant disease with increasingly raising incidence.

## 2. Results

**Figure 2:**
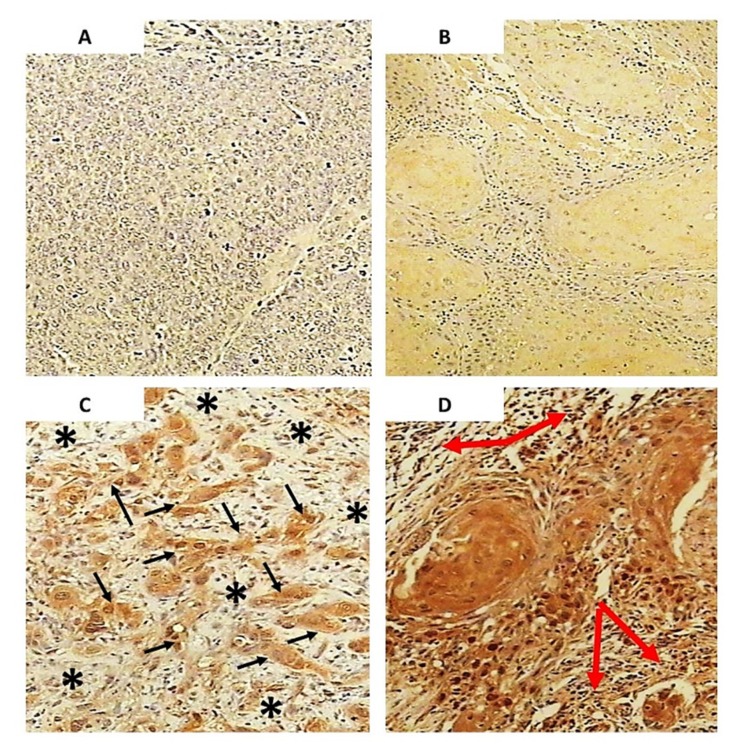
The immunohistochemical appearance of HNE in squamous cell carcinoma of the oropharynx. (**A)** Control staining without monoclonal antibody against HNE-protein adducts (consequently there is no brown chromogen reaction of DAB). (**B)** The appearance of the HNE-modified proteins mostly in cytoplasm of tumor cells of carcinoma grade I expressed at low staining intensity presented as yellow/light brown color (low magnification). (**C**) The appearance of the HNE-modified proteins carcinoma of the grade III mostly in cytoplasm and in some nuclei of tumor cells present as low to moderate intensity (some examples are indicated by small black arrows). Surrounding stroma is negative for HNE (indicated by asterisk), while chronic inflammatory cells, presented as dark spots, show presence of HNE (high magnification). (**D**) The appearance of HNE-modified proteins in another tumor of grade III but with moderate to high intensity of immunostaining. Surrounding stroma and chronic inflammatory cells (indicated by big red arrows) also show the presence of HNE.

The study included 102 patients operated on for oropharyngeal squamous cell carcinoma. The intensity and distribution of HNE-protein adducts in tumors and in non-tumorous oropharyngeal tissue were studied. The presence of HNE-protein adducts in tumor cells (Table 1, Figure 2) was detected in almost all samples (99/102), confined mostly to the cytoplasm of cancer cells.

The HNE-immunopositivity was semi-quantitatively graded as +1 in 5 samples, +2 in 8 samples and +3 in 86 samples (according to the incidence of immunopositive cells), while immunostaining of low intensity was found in 50 samples, moderate intensity in 40 samples and strong intensity in 9 samples (according to the brown color appearance upon the DAB staining). Interestingly, even in patients whose cancer cells were mostly immunopositive for HNE, only a few had strong intensity of immunostaining (8/86).

The nuclei of cancer cells were mostly negative for HNE (69/102), while in the case of positive immunostaining its intensity was very strong and frequently present in cancer cells of only eight patients, i.e., in the same specimens that were also expressing the highest immunopositivity of the cytoplasm of malignant cells.

**Figure 3:**
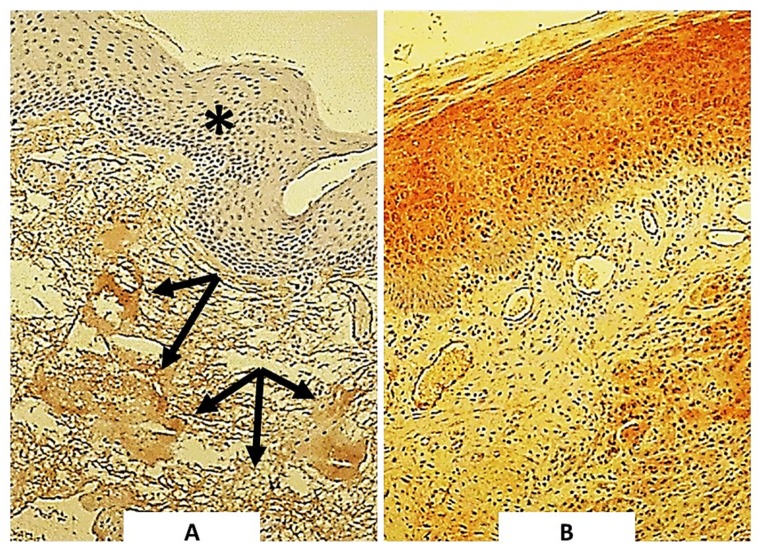
The immunohistochemical appearance of HNE in normal, healthy oropharyngeal mucosa. (**A**) No HNE can be seen in the normal epithelium of the healthy subject (indicated by asterisk), although adjacent stromal tissue contains HNE (indicated by arrows). (**B)** Prominent presence of HNE in non-malignant epithelium in the vicinity of squamous cell carcinoma grade III of the oropharynx.

The presence of HNE-protein adducts in the normal cells within tumor and surrounding tissue is presented in Figure 3 and Table 2.

Mesenchymal stroma was positive for HNE in less than half of the cases (41/97), while blood vessels (walls) were HNE-immunopositive in more than half of the cases (58/97). Opposite to that, endothelium of blood vessels was negative for HNE in the majority of the cases (87/97). As expected, if present, inflammatory cells, notably lymphocytes and plasma cells, contained HNE in majority of the cases (66/79).

**Figure 4:**
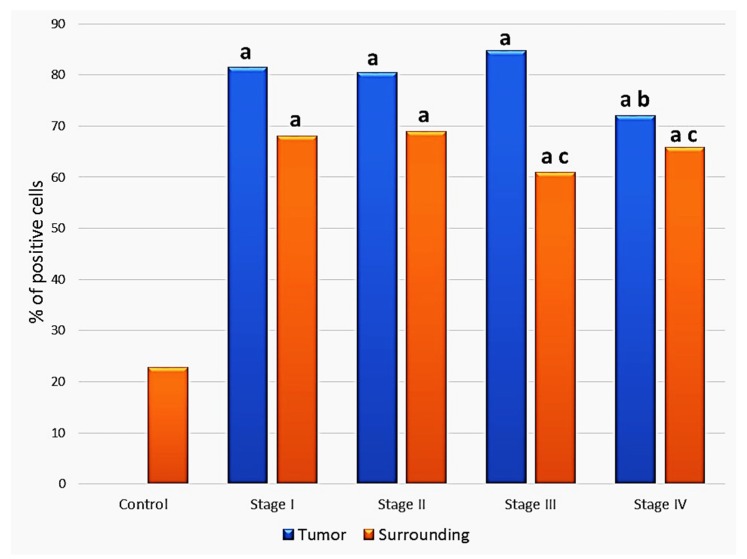
Comparison of the HNE immunopositivity in the cytoplasm of squamous carcinoma cells and in non-malignant epithelial cells adjacent to the tumor depending on the clinical stage of the disease. a—significance to the healthy controls subjects; b—significance to the cancer cells of the stage III carcinoma specimens; c—significance to the non-malignant cells surrounding cancer tissue graded as stage I or II.

As can be seen in Figure 4, the HNE-immunopositivity in the epithelium close to carcinoma was elevated in non-malignant cells of all clinical stages of carcinoma in comparison to the control group, without cancer (*p* <0.05). A significant difference was also found for malignant cells by comparison between stages III and IV (*p* = 0.038).

In non-malignant epithelial cells close to the tumor intensity of staining was higher in advanced malignancies. Hence, a statistically significant difference was eventually found comparing the mean values between clinical stages II and III (*p* = 0.042) and clinical stages II and IV (*p* = 0.012).

## 3. Discussion

The aim of this study was to determine the immunohistochemical expression of HNE-histidine conjugates, as biomarker of OS in squamous cell carcinoma of the oropharynx. Our findings indicate that HNE is differentially distributed in squamous cell carcinomas in dependence of clinical stage and histological grading of these tumors. The tonsils and the basis of the tongue represented about 90% of all oropharyngeal squamous cell carcinomas in our study, thus resembling usual appearance of these malignances [28,29]. The stage of the disease at the time of diagnosis is among the most accepted prognostic factors. More than half of the patients involved in this study (61.7%) were in advanced stages of the disease. The second prognostic factor is the histological grade of tumor differentiation that cannot be associated with the probability of metastasis and does not show a uniform distribution in the stage of the disease.

In our study, two out of three of the patients had moderate to poorly differentiated tumors, which were at the advanced stages of the disease. The appearance of LPO according to the presence of HNE-protein adducts was found in tumor cells of almost all analyzed squamous cell carcinomas. Alongside the cancer cells, the HNE-protein adducts were found within the wall of the blood vessels, tumor stroma and inflammatory cells of most analyzed samples.

Therefore, our findings show the onset of OS in squamous cell carcinoma of oropharynx, which is associated with LPO and the consequent synthesis of HNE, being higher in tumor cells than in the surrounding non-tumor epithelium and in the control samples. The level of HNE immunopositivity increased in well and moderately differentiated squamous cell carcinoma, while it was gradually decreasing in poorly differentiated carcinomas and in advanced stages of disease.

The presence of HNE was found at a high percentage in cytoplasm of tumor cells in all tumor grades as well as in the surrounding epithelium close to carcinoma, while the presence of HNE in cell nuclei was found to be in a lower percentage. In the control group, HNE positivity was found only in the cytoplasm of cells, whereas the nuclei in all samples were negative. By comparing the clinical stages of disease, HNE positivity was more pronounced in cytoplasm than in nuclei in tumor cells and fell in stage IV of disease compared to the first three stages; it was statistically significant only for stage III of the disease. The intensity of HNE immunoreaction in the first three stages of the disease was generally moderate; at stage IV of the disease, the intensity was only weak. At all stages of the disease, expression of HNE in the surrounding non-tumor epithelium was positive in cytoplasm of cells in relation to the control group, particularly in cases of stages II and IV of the disease.

The presence of HNE-protein adducts was also found in inflammatory cells, especially macrophages, in the stroma of the tumor and around tumor. It has been observed that the level of HNE in stroma decreased with tumor grade, while HNE-positivity in tumor stroma was stronger than in control, healthy tissue. This can be explained by the fact that inflammatory cells produce a large amount of ROS and thus induce lipid peroxidation. In support of this, the presence of HNE positivity in tumor stroma and inflammatory cells was related to the presence of HNE in tumor cells depending on the carcinoma degree. During the phagocytosis, there is an “oxidative burst” of inflammatory cells producing ROS, causing further enhancement of OS and LPO [30]. The increase of ROS is associated with disturbed tumor cell growth and is a homeostasis disorder that is caused either by increased ROS generation or by reduced ROS-enzyme elimination [31]. It has been confirmed that levels of enzymes removing ROS such as SOD, glutathione peroxidase and peroxyredoxin are significantly impaired in malignant cells and tissue, all leading to impaired homeostasis and stress adaptation in tumor cells [32]. This can lead to a *circulus vitiosus* where reactive aldehydes can be only partially removed from the tissue. However, the accumulated HNE can cause death of the tumor cells. To avoid that, cancer cells may contain lowers amounts of lipids and PUFAs, thus reducing the production of HNE in the tissue [32]. This can explain the reduced amount of reactive aldehydes in advanced stages compared with earlier stages of the disease, resembling also results obtained for the primary hepatocarcinomas [33,34]. Such a similarity between different types of cancer is in agreement with findings that were already obtained in the 1960s, showing that the different malignant cells express similar patterns of altered lipid metabolism, consequently resembling each other more than resembling their respective, non-malignant counterpart cells [35]. Therefore, it is not surprising that oropharyngeal carcinomas analyzed in the current study are similar to findings obtained for liver cancer. The process of liver carcinogenesis analyzed on particular strains of rats (LEC) that accumulate copper, acting as a pro-oxidant transition metal, appears to be associated with a similar process of gradual increase of HNE-protein adducts, which correlates to the onset of jaundice and hepatitis [36]. Interestingly, although non-malignant cells only rarely express positive immunostaining for the HNE-protein adducts in the nuclear part of the cells, in the case of liver carcinogenesis occurring in the LEC rats, nuclei of some cells were HNE-positive even before jaundice, while the nuclear immunopositivity for HNE was lost afterwards. Eventually, thus transformed hepatocarcinomas cells did not contain HNE-protein adducts at all, while the non-malignant liver cells in their vicinity were loaded with HNE-protein adducts [34], Therefore, we think that such dynamic changes could be responsible for the observed differences between the onset of the HNE-immunopositivity in the cytoplasm and in the nuclei of oropharyngeal carcinoma cells, as observed in the current study, keeping in mind that each cancer is a specific entity and every patient is a different individual.

Similarly, the study of colon adenocarcinoma revealed the reduced presence of another lipid peroxidation product acrolein in advanced cancer [37]. In this study, different presence of acrolein in benign adenomas and in malignant adenocarcinomas of the colon were observed, depending on the clinical stage of the disease and the histological tumor grade, eventually being lower in advanced than in the earlier stages of the disease. However, the expression of acrolein was found to be abundant in non-malignant colon epithelium surrounding advancing cancer tissue, thus resembling findings obtained for human and murine liver malignancies [33,35]. These results should be interpreted considering the complex pattern of HNE-metabolizing enzymes in tumor cells; the lipid composition of the cell membranes with a different level of peroxidizable substrates in normal and in malignant cells (such as PUFAs); and the presence of inflammatory cells, which can increase the level of diffusible HNE, acrolein and related lipid peroxidation products from the tumor-surrounding tissues, especially if bound to the proteins [32].

The relevance of inflammatory cells and tumor stroma in the onset of LPO during carcinogenesis and in the host defense against invading cancer were described both in clinical trials and experimental models of murine cancer regression. Namely, the phenomenon of spontaneous regression of W256 murine tumors was found to be associated with pronounced oxidative bust and anticancer activities of neutrophil granulocytes generating HNE and acrolein, resembling the effects of granulocytes and HNE on melanoma B16 [38,39,40], while in patients with metastatic lung cancer the onset of inflammation related to the invading metastatic cancer and the accumulation of HNE-protein adducts were found to be more pronounced than in the case of primary lung cancer [41].

On the other hand, changes of lipid metabolism during carcinogenesis and cancer spread in the organism certainly do depend also on the type of cancer, even if originating from the same organ, reflecting high individual differences even between the same types of malignant cells, as was observed for the human lung cancer [42]. Consequently, these will result also in a differently expressed onset of LPO, not only on the level of the affected non-malignant tissue and cancer itself, but also on the level of the entire organism, similarly to the other metabolic changes caused by cancer [43].

Finally, together with the above mentioned involvement of HNE in the innate immune system anti-cancer effectiveness, a possible crucial role of HNE in the host defense against cancer might be based on its universal regulatory activities affecting lipid metabolism and the growth of the cells on one hand, and on the other, the selective anti-cancer effects of HNE, depending on its antioxidant, pro-apoptotic and cytokine signaling regulation, proportional to its binding to the (extra)cellular proteins [44,45,46,47,48,49]. Among these, recently revealed selective anticancer effects of HNE generated by the non-malignant cells attenuating or even entirely blocking cancer-specific tumor membrane-associated catalase, might be of particular relevance [50].

In conclusion, this study revealed the onset of OS and LPO in the development of human malignant epithelial tumors and the immunohistochemical presence of HNE in squamous cell carcinoma of oropharynx and in surrounding non-tumorous tissue. Our study also further supports the assumption of HNE as a bioactive marker of OS and a potentially defensive, anti-cancer factor generated by surrounding non-malignant cells.

## 4. Materials and Methods

### 4.1. Tumors

We analyzed 102 surgical specimens diagnosed as oropharyngeal squamous cell carcinoma at the Department of Pathology and Cytology, University Hospital Center Zagreb, during the period 2002 to 2007. The mean age of patients was 59 years, and the group consisted of 11 females and 91 males. The youngest participant was 21 years old, whereas the oldest one was 88 years old. They included 51 cases of tongue base carcinoma, 38 cases of tonsil carcinoma, 7 cases of posterior pharyngeal wall carcinomas, and 6 cases of soft palate carcinoma. All the tumors were classified according to the criteria of WHO [12] for histological grades, 27 cases of SCC grade I, 40 cases of SCC grade II, and 35 cases of SCC grade III. For all patients, pathohistological data (tumor size, histologic type of SCC, histologic grade, lymph node status, and distant metastasis) were obtained. Based on TNM classification, tumors grade T1 and T2 are classified as early, while T3 and T4 are advanced. The analyzed sample included 58 early diagnosed tumors and 44 locally advanced tumors. According to American Joint Committee on Cancer (AJCC), this study included 39 cases of early stages (stage I and II) of disease and 63 cases of advanced (stage III and IV) stages of disease. Ethical Committee of the Clinical Hospital Centre Zagreb approved the study under permit name “Metabolomic and pathomorphological trials in oncology” and 02/21 AG number. 

### 4.2. Normal Oropharyngeal Tissue

The control group consisted of 32 samples of oropharynx mucosa tissue operated after tonsillectomy due to chronic tonsillitis or oropharynx mucosa samples obtained according to the protocol for diagnosis of tumors of an unknown primary location. In all control samples, histopathological examination showed no tumor or other inflammatory and reactive changes.

### 4.3. Tissue Processing

All tumors used in the study were surgically resected at Department of ENT surgery, University Hospital Center Zagreb. The specimens were fixed in 10% buffered formalin immediately after resection, dehydrated in ethanol, and embedded in paraffin. Representative paraffin blocks of each tumor and surrounding mucosa were cut in three 5-µ-thin slices examined by section staining with hematoxylin and eosin and immunohistochemistry method using monoclonal antibody for HNE-histidine conjugates. Immunohistochemical staining was performed using a monoclonal antibody for the detection of HNE-modified proteins. It was obtained from the culture medium of the clone derived from a fusion of Sp2-Ag8 myeloma cells with B-cells of a BALBc mouse immunized by HNE-modified keyhole limpet hemocyane [51]. The antibody is specific for the HNE-histidine epitope in HNE-protein (peptide) conjugates. Dilutions of antibody solution and appropriate reagents from the EnVision detection kit (K 8000, DAKO) were used on a DAKO automated immunostainer. Antigens were localized using an avidin-biotin method with 3, 3^,^-diaminobenzidine (DAB) as a chromogen, and counterstained with hematoxylin (Kemika, Zagreb, Croatia). The immunohistochemical investigation of intensity and distribution of HNE in the tumor and surrounding non-tumorous mucosa were determined and scored in a semi-quantitative way (0: 0% positive cells, +1: 1–25% positive cells, +2: 26–50% positive cells, +3: 51–100% positive cells). Staining intensity was divided into three groups: low, moderate and strong. The presence of HNE-protein adducts in specific structures like endothelial cells and walls of blood vessels, mesenchymal stroma, and chronic inflammatory infiltrate was defined as negative (−) in the absence of the HNE-protein adducts and as positive (+) in the presence of HNE-protein adducts. Two pathologists diagnosed each specimen independently.

### 4.4. Statistical Analyses

The incidence of HNE-positive vs. HNE-negative tissues depending on the type of tumor was evaluated by chi-square test. Possible differences in the intensity of staining were examined by Mann-Whitney test, using a numerical description of positivity corresponding to the respective standard grading of positivity as described above.

## Figures and Tables

**Table 1:** Semiquantitative analysis of the HNE-immunopositivity and intensity of immunochemical reaction in malignant cells.

HNE-Immunopositivity	Cytoplasm of Tumor Cell		Nuclei of Tumor Cell	
	No. of Specimens/Patients	Intensity of Immunostaining *	No. of Specimens/patients	Intensity of Immunostaining*
0	3	-	69	-
1	5	50	17	9
2	8	40	4	16
3	86	8	12	8

Legend: ′0′ negative—cancer cells were not positive for HNE, ′1′ HNE-immunopositivity was observed in less than 25% of tumor cells, ′2′—immunopositivity was observed in 25–50% tumor cells, ′3′ immunopositivity was observed in more than 50% of cancer cells; *—according to the brown color developed using 3, 3,-diaminobenzidine (DAB) chromogen.

**Table 2:** The presence of HNE-immunopositivity in non-malignant tissue cells within/near tumor.

HNE-Positivity	N *
**Stromal cells**	
Positive	41
Negative	56
**Blood vessel wall**	
Positive	58
Negative	39
**Endothelium**	
Positive	10
Negative	87
**Inflammatory cells**	
Positive	66
Negative	13

*—number of patients/specimens analyzed (the remaining up to 102 in total did not contain the respective type of cells/tissue).
